# Outbreak of *Salmonella* Newport linked to imported frozen cooked crayfish in dill brine, Sweden, July to November 2019

**DOI:** 10.2807/1560-7917.ES.2022.27.22.2100918

**Published:** 2022-06-02

**Authors:** Marie Jansson Mörk, Nadja Karamehmedovic, Anette Hansen, Joanna Nederby Öhd, Mats Lindblad, Emma Östlund, Moa Rehn, Cecilia Jernberg

**Affiliations:** 1 Public Health Agency of Sweden, Solna, Sweden; 2 European Programme for Intervention Epidemiology Training (EPIET), European Centre for Disease Prevention and Control (ECDC), Solna, Sweden; 3 County Council Department of Communicable Disease Control and Prevention, Stockholm Region, Stockholm, Sweden; 4 Swedish Food Agency, Uppsala, Sweden; 5 National Veterinary Institute, Uppsala, Sweden

**Keywords:** Salmonella, case-case study, outbreak, crayfish

## Abstract

In autumn 2019, the Public Health Agency of Sweden identified a cluster of *Salmonella* Newport cases by whole genome sequencing (WGS). Cases’ distribution in place and time indicated a nation-wide ongoing outbreak. An investigation was initiated to identify the source and prevent further cases. We conducted a case–case study based on notified salmonellosis cases and a *Salmonella* trawling questionnaire, comparing 20 outbreak cases and 139 control cases. Food exposures were compared by adjusted odds ratios (aOR) with 95% confidence interval (CI) using logistic regression. Implicated foods were sampled. Outbreak cases were more likely to have consumed crayfish (aOR = 26; 95% CI: 6.3–105). One specific brand of imported frozen, pre-cooked whole crayfish in dill brine was identified as the source. *Salmonella* Newport was later detected in different batches from retail and in one sample from border control. Isolates from food samples clustered with the human outbreak strain by WGS. Although the retailer made a complete recall, two more cases were identified long afterwards. This investigation demonstrated the successful use of a case–case study and targeted microbiological testing to identify the source. The immediate action taken by the retailer was important to confirm the source and stop the outbreak.

## Background

Salmonellosis is the second most commonly reported bacterial gastro-intestinal infection after campylobacteriosis in the European Union (EU) and European Economic Area (EEA). In the EU/EEA, the yearly notification rate of salmonellosis has been around 20 cases per 100,000 individuals in the period from 2015 to 2019 [1]. In 2020, the notification rate decreased to 14 cases per 100,000 individuals; the decline is considered to be an effect of the COVID-19 pandemic [2]. *Salmonella enterica* serotype Newport was the fifth most common serotype in the EU/EEA in 2019, accounting for 1.1% of reported serotypes from human salmonellosis cases [1]. In Sweden, human salmonellosis is notifiable by law. The average yearly notification rate of salmonellosis in Sweden between 2015 and 2019 was 21.5 cases per 100,000 individuals, with the majority of cases being infected abroad [3]. In 2019, *Salmonella* Newport was the fifth most common serotype also in Sweden and accounted for 5% of the reported serotypes [3].

Salmonellosis is a bacterial zoonosis. Humans get infected by contaminated foods, through contact with infected animals or humans or via the environment [4]. In recent years, egg and egg products, bakery products and pork and pork products have been the most common food vehicles in food-borne salmonellosis outbreaks reported in EU countries [1]. *Salmonella* Newport has previously been linked to outbreaks caused by vehicles of both animal and vegetable origin, for example beef, watermelon and mung bean sprouts [5-8]. *Salmonella* Newport have also been detected in fish and different shellfish and in fresh herbs [9-11].

## Outbreak detection

In late September 2019, the Public Health Agency of Sweden (PHAS) identified a cluster of nine cases with *Salmonella* Newport sequence type (ST) 46. The cluster was detected as part of the routine microbial surveillance programme where isolates of *Salmonella* from domestic infections are sent to PHAS for typing using whole genome sequencing (WGS). The cluster was put under observation; it evolved slowly and on 23 October, the cluster consisted of 25 cases. The cases were geographically spread across the country and all but one case were adults. The onset of disease ranged from 16 August to 12 October 2019. The spread of cases, geographically and in time, indicated that the infection source was a contaminated food that was distributed nation-wide and could still be on the market. A national outbreak was declared and an outbreak investigation was initiated with the objectives to describe the outbreak and identify the source in order to prevent further cases.

The outbreak team included investigators from PHAS, the Swedish Food Agency (SFA) and the affected regional Departments of Communicable Disease Control and Prevention (CDC department).

Here we report an investigation of a national outbreak of *Salmonella* Newport in Sweden, with the aim of describing the actions that led to the identification and recall of the source of the infection. 

## Methods

### Outbreak case definition

A confirmed case was defined as a domestic case of *Salmonella* infection notified in SmiNet (the Swedish notification system for notifiable communicable diseases) from 19 August 2019 to 13 November 2019 and with a *Salmonella* Newport ST46 isolate clustering by a maximum of five single nucleotide differences (SNPs) using WGS.

### Epidemiological investigation

Information regarding age, sex (male/female), county of residence, date of symptom onset and sampling date of the cases was obtained from SmiNet.

#### National *Salmonella* trawling questionnaire

The national *Salmonella* trawling questionnaire is a web-based questionnaire. It is used in routine investigation of notified cases by the regional CDC departments and responses are shared with PHAS for use in national outbreak investigations. The questionnaire collects information about symptoms relevant for *Salmonella* infection, demographical data and exposures during the period 7 days before onset of disease, i.e. travel in Sweden and abroad, visits to restaurants or cafés, consumption of ready-to-eat food and specific foods, water supply, contact with pets or farm animals, gardening and contact with wild game at slaughter. The exposure questions are closed-ended questions with three (yes, no or don’t know) or four (yes, probably, probably not and no) options and with additional open-ended questions about brand and place of purchase or specific type of foods.

#### Case–case study

At the start of the investigation, most cases had been interviewed by the CDC departments and 11 cases had also responded to the national *Salmonella* trawling questionnaire. Several cases stated that they had eaten crayfish. This was, however, not unexpected since crayfish feasts are a traditional celebration in Sweden in August and September. In order to investigate the hypothesis that crayfish or dishes common at crayfish feasts were the source of the outbreak, we conducted a case–case study using data collected through the national *Salmonella* trawling questionnaire from 2017 to 2019.

We used domestic salmonellosis cases, notified in SmiNet, with infections with known *Salmonella* serotype as control cases. Cases eligible as controls were younger than 4 or older than 19 years, to match the ages of the outbreak cases, and had onset of disease from 15 August to 31 October 2017, 2018 or 2019. Thus, cases with serotype Newport but not meeting the outbreak case definition as well as cases linked to other outbreaks were eligible as control cases. Also, cases linked via WGS to a simultaneously ongoing outbreak of monophasic *Salmonella* Typhimurium were excluded as controls [12].

We included responses from outbreak and control cases who completed the whole questionnaire in the analysis. Foods to which at least 30% of the cases were exposed were included in the analysis. The options 'yes' and 'probably' were categorised as exposed and 'no' and 'probably not' were categorised as unexposed. The option 'don’t know' was categorised as missing.

Based on the additional open-ended question, information about consumption of shellfish and mushrooms were further categorised into crayfish and prawns, and mushrooms and chanterelles, respectively. Similarly, specific retail companies where the cases bought their food were categorised based on additional open-ended questions about place of purchase. Age was categorised into three groups (children ≤ 3 years, adults 20–59 years and adults ≥ 60 years). Region was categorised as South (Blekinge, Skåne, Halland, Västra Götaland, Jönköping, Kalmar, Kronoberg, Gotland and Östergötland), Centre (Värmland, Dalarna, Gävleborg, Uppsala, Stockholm, Södermanland, Västmanland and Örebro) and North (Norrbotten, Västerbotten and Västernorrland).

#### Statistical analyses 

Statistical analyses were performed in Stata (version 16.0, Stata Corp., College Station, Texas, United States). We used logistic regression, with outbreak/control case (1/0) as the dependent variable. We estimated adjusted odds ratios (aOR) with 95% confidence intervals (CI) and p values. Initially, each exposure was tested separately, adjusting for age group, region and sex. All exposures with a p value < 0.2 were included in the multivariable analysis. The full model was reduced using manual backward elimination until all remaining exposures had a p value < 0.05, which was set as the threshold for significance. The adjusting variables age group, region and sex were kept in the final model regardless of statistical significance to adjust for different distribution between outbreak cases and control cases. All exposures excluded from the final model were analysed stratified by each exposure remaining in the final model to check for effect modification. If the stratum specific odds ratios (OR) differed, the stratifying exposure was considered to be an effect modifier and stratified analyses were conducted. Also, to check for confounding, the effect of excluded exposures from the final model on the remaining exposures was investigated. If the OR changed with more than 10%, the excluded exposure was deemed a confounder and included in the final model.

#### Additional questionnaire

Based on the findings from interviews with the cases and the responses to the trawling questionnaire, PHAS compiled additional questions. They included details about brand, country of origin and food chain for crayfish and liver pâté as well as side dishes, garnish and sauces commonly served with crayfish. This was done in order to distinguish specific brands for the foods identified as possible vehicle of infection in the case–case study, and to identify any common ingredient eaten with crayfish. This additional information was retrieved through telephone interviews done by the regional CDC departments.

### Microbiological analysis of food

Analyses of samples from foods were performed by commercial laboratories. In one region, a municipal environment and health team collected samples from a grocery store. The retail company selling the implicated food managed the sampling of the product in their storage and gave the authorities access to isolates from *Salmonella*-positive samples. Further microbiological investigation of *Salmonella*-positive samples with WGS was performed at the National Veterinary Institute. Samples were prepared with an Illumina Nextera XT Library Preparation Kit (Illumina Inc) and sequencing was performed with an Illumina MiSeq (Illumina Inc) producing 250 bp paired-end reads. Reads were trimmed with Trimmomatic v. 0.39 [13] and assembled with Unicycler v. 0.4.8 [14]. Sequence type was determined with the Achtman seven-gene multilocus sequence typing (MLST) scheme using mlst v. 2.11 and the PubMLST database [15,16]. A SNP analysis was performed to confirm that the food isolates clustered with the human isolates. Sequence data from two isolates were provided by PHAS for this analysis. Trimmed reads were mapped to a *Salmonella* Newport reference genome (GenBank accession number: CP012598.1) using Bowtie2 v. 2.3.5 [17] and SNPs were called and filtered using BCFtools v. 1.8 [18] and manually filtered using Integrated Genomics Viewer v. 2.4.17 [19].

Raw read sequences were shared with PHAS to be compared with all the human isolates from the current outbreak by SNP analysis.

### Microbiological investigations of human isolates and comparison of human and food isolates

Isolation of *Salmonella* spp. in clinical specimens was routinely performed by local clinical microbiological laboratories. Isolates were sent to PHAS for further microbiological investigation using WGS as part of microbiological surveillance programme at PHAS. The Ion Xpress Plus Fragment Library Kit for AB Library Builder System (Thermo Fisher Scientific), was used for library preparation and sequencing was performed with Ion S5-XL System (Thermo Fisher Scientific). The expected mean read length was 300 bp, and the minimum average coverage depth 20 ×. Sequence type (ST) was determined using the Achtman seven-gene MLST scheme from Enterobase [20,21], and serotype prediction was performed in silico using SeqSero [22] and an in-house database of serotypes and STs. To predict antimicrobial resistance genes and point mutations, assembled genomes were analysed using StarAMR (with a genome size cut-off of 4.6–5.4 Mbp and PointFinder set to detect *Salmonella* mutations) to acquire ResFinder and PointFinder results for the genomes [23]. Clustering and comparison of human and food isolates were performed by SNP analyses, with the pipeline used as part of the routine clustering analysis at PHAS. To perform the SNP analysis, a single representative sequence from the outbreak was used as a reference and the SNP analysis was visualised in a minimum spanning tree generated with MSTgold [24].

## Results

### Descriptive epidemiology

**Figure 1:**
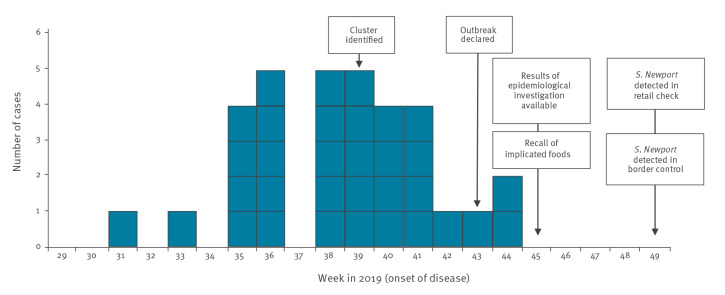
Number of confirmed outbreak cases by date of onset of disease, and timeline of the investigation, *Salmonella* Newport outbreak, Sweden, July–December 2019 (n = 33)

In total, 33 cases were confirmed, with onset of symptoms between 31 July 2019 and 2 November 2019 (Figure 1). Cases were distributed among 12 of the 21 counties in Sweden, with between one and eight cases in each region (median: two cases). Cases had a mean age of 53 years (range: 1–82 years) and the majority were female (n = 20).

### Case–case study

Twenty outbreak cases completed the questionnaire and 139 other cases fulfilled the criteria for control cases. The distribution of outbreak cases and control cases included in the case–case study per region, age category and sex are shown in Table 1. Control cases included 53, 36 and 50 cases from 2017, 2018 and 2019, respectively. 

**Table 1:** Number and percentage of outbreak cases (n = 20) and control cases (n = 139) per age category, region and sex, case–case study using the Swedish *Salmonella* trawling questionnaire, Sweden, July–November 2019

Case characteristic		Outbreak cases		Control cases	
		n	%	n	%
Age category	≤ 3 years	1	5	9	6
	20–59 years	8	40	92	66
	≥ 60 years	11	55	38	27
Region	South of Sweden	4	20	86	62
	Centre of Sweden	13	65	45	32
	North of Sweden	3	15	8	6
Sex	Female	13	65	77	55
	Male	7	35	62	45

The exposures investigated in the study are shown in Table 2. The multivariable analysis showed that the outbreak cases were more likely to have consumed crayfish compared with the control cases (aOR = 26; 95% CI: 6.3–105) (Table 2). The exposure 'dish with raw eggs' was identified as a confounder and kept in the model although not statistically significant.

**Table 2:** Number and percentage exposed, adjusted odds ratios of outbreak cases (n = 20) compared with control cases (n = 139), *Salmonella* Newport outbreak investigation, Sweden, July–November 2019

Exposure	Outbreak cases (n = 20)		Control cases (n = 139)		Model for each exposure^a^			Final model^b^		
	n	%^c^	n	%^c^	aOR	95% CI	p value	aOR	95% CI	p value
Crayfish	15	75	12	8.8	32	8.0–128	< 0.001	26	6.3–105	< 0.001
Dish with raw eggs	7	37	20	15	4.7	1.4–16	0.01	2.3	0.5–10	0.28
Liver pâté	9	47	25	20	4.0	1.3–12	0.02	Not included		
Mushrooms	6	30	17	12	3.9	1.1–14	0.04			
Blueberries	6	35	16	13	3.4	1.0–12	0.05			
Roasted chicken	6	30	19	15	3.4	0.9–13	0.08			
Grapes	8	47	45	35	2.5	0.8–8.0	0.14			
Minced meet (mix of beef and pork)	8	44	47	36	2.3	0.7–7.2	0.15			
Ready-to-eat meals	6	30	23	17	2.3	0.7–7.4	0.17			
Dill	6	32	17	13	2.2	0.7–7.3	0.21			
Chicken, other	8	47	43	33	2.1	0.7–6.3	0.19			
Minced meat (beef)	11	55	63	47	2.0	0.6–6.0	0.24			
Cabbage	7	35	28	23	1.9	0.6–5.9	0.26			
Prawns	7	35	36	26	1.5	0.5–4.5	0.49			
Lettuce (all sorts)	12	60	64	49	1.4	0.5–4.1	0.50			
Pepper	11	55	55	43	1.4	0.5–4.2	0.50			
Broccoli	6	30	32	25	1.3	0.4–4.0	0.69			
Muesli	6	30	35	26	1.3	0.4–4.1	0.68			
Tomato	16	80	94	72	1.1	0.3–3.9	0.92			
Spinach	7	35	39	30	1.1	0.4–3.4	0.83			
Cashew nuts	6	30	31	24	1.1	0.3–4.0	0.83			
Cucumber	15	75	99	75	0.9	0.3–3.0	0.85			
Smoked ham	10	53	64	50	0.9	0.3–2.4	0.77			
Carrots	10	50	60	46	0.9	0.3–2.6	0.84			
Apples	10	50	68	52	0.9	0.3–2.7	0.87			
Lightly smoked bologna sausage	9	45	46	35	0.9	0.3–2.6	0.81			
Iceberg lettuce	8	42	57	44	0.9	0.3–2.6	0.82			
Bacon	7	37	48	37	0.9	0.3–2.6	0.82			
Yellow onion	12	60	77	60	0.8	0.3–2.4	0.70			
Peanuts	6	30	34	26	0.8	0.3–2.5	0.72			
Red onion	8	40	59	47	0.7	0.3–2.2	0.59			

aOR: adjusted odds ratio; CI: confidence interval.

^a^ Each exposure adjusted for sex (female^d^ and male, age group (≤ 3 years, 20–59 years^d^, ≥ 60 years) and region (south^d^, centre and north of Sweden).

^b^ Final model, reduced full model by manual backward elimination of exposures, adjusted for sex (female^d^ and male), age group (≤ 3 years, 20–59 years^d^, ≥ 60 years), region (south^d^, centre and north of Sweden) and the other exposure in the model.

^c^ Percentage exposed of those who answered that question.

^d^ Reference group; unexposed was the reference group for food exposures.

### Additional questionnaire

From the additional questions about foods and the open-ended questions in the trawling questionnaire, we identified one specific brand of frozen, pre-cooked whole crayfish in dill brine imported from China.

### Microbiological analysis of food

The results of the epidemiological investigation were completed and communicated to the retail company selling the specific brand of crayfish in dill brine on 8 November. The retail company recalled all remaining packages immediately. From the recalled batches, with production date from 30 November 2018 to 4 December 2018, the retail company analysed 84 samples from different batches. *Salmonella* Newport was detected in six samples from batches with best-before date 1 and 2 December 2021, respectively. Also, *Salmonella* Newport was detected in the same brand of crayfish in dill brine in follow-up sampling performed at one of the SFA’s border control posts. The best-before date for this positive batch was 12 December 2021. Samples from packages of crayfish in dill brine collected from a store by the municipal environment and health team were negative for *Salmonella*.

### Whole genome sequencing of human and food isolates

**Figure 2:**
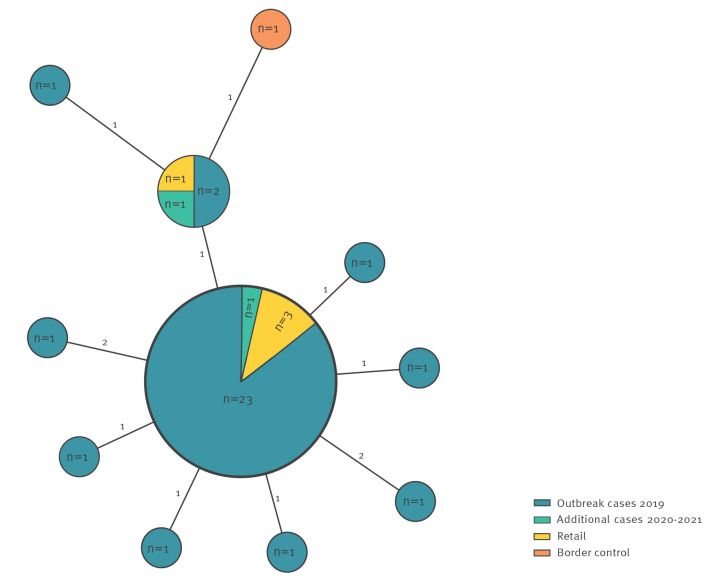
Minimum spanning tree of *Salmonella* Newport ST46 isolates from outbreak cases (n = 33) and food (n = 5), Sweden, July–November 2019

All isolates, both of human and food origin, belonging to the outbreak profile *Salmonella* Newport ST46 clustered within 0–5 SNP differences (Figure 2). The SNP analysis was used to cluster the combined IonTorrent and Illumina reads from human and food isolates which is part of the routine analysis pipeline at PHAS. Sequences from two of the six isolates from retail did not pass the quality parameters and are therefore not included in the cluster analysis.

Single nucleotide polymorphism differences are shown next to the branches and branch lengths are not proportional to genetic distance. One single representative sequence from the outbreak was used as a reference. Tree colours are based on isolate source. Two additional isolates, one in 2020 and one in 2021, identified after the outbreak, are also included.

None of the outbreak isolates, of human or food origin, had markers for predicted antibiotic resistance.

The PHAS WGS database showed that *Salmonella* Newport ST46 had only been identified twice in Sweden before the onset of this outbreak, in one isolate from a case infected in 2017 and in one isolate from the beginning of 2019. The SNP analysis showed that the two historical isolates were not related to the outbreak strain. Three other STs were identified in Sweden during 2019: ST31, ST45 and ST118.

## Outbreak control measures

The PHAS posted information about the outbreak on their website and the retail company of the incriminated product issued a press release about the withdrawal. To enquire about cases in other countries, PHAS posted an urgent inquiry (UI-609) on the European Centre for Disease Prevention and Control (ECDC) Epidemic Intelligence Information System for Food and Waterborne Diseases and Zoonoses (EPIS-FWD) on 29 October 2019. Twelve countries responded to the inquiry and none had experienced an increase in *Salmonella* Newport. On 3 and 5 December, the SFA posted a notification in the Rapid Alert System for Food and Feed (RASFF) to inform competent authorities in the exporting country through the International Food Safety Authorities Network (INFOSAN), in order for them to follow up with the producer (Reference number 2019.4248 and 2019.4269).

Two additional cases with the outbreak strain were later identified, one in 2020 and one in 2021. The case in 2020 had eaten crayfish from one of the recalled batches in 2019, whereas the case in 2021 claimed not to have eaten crayfish before disease onset.

## Discussion

The epidemiological investigation pointed out a specific imported brand of frozen cooked whole crayfish in dill brine as the suspected vehicle of *Salmonella* Newport ST46 infection. The vehicle of infection was later confirmed microbiologically in samples from recalled batches of crayfish and in a sample from border control. Despite the complete recall of the product, two additional cases with the outbreak strain were identified, one in 2020 and one in 2021. This demonstrates the difficulty of completely removing the risk associated with contaminated foods with long best-before dates, as the chance of connecting a food in the freezer with a recall, or news of an outbreak, decreases over time. 

This is, to our knowledge, the first *Salmonella* outbreak linked to frozen pre-cooked crayfish in dill brine. Outbreaks of *Salmonella* Newport have previously been linked to both animal and vegetable vehicles, for example beef [5,6], watermelon [7] and mung bean sprouts [8]. It is not known how the product was contaminated. *Salmonella* has previously been detected in wild crayfish [25] and it is possible that the crayfish were undercooked. However, since *Salmonella* was detected in two batches, indicating a persistent contamination, it is more likely that the product was contaminated after cooking, by adding contaminated dill or by environmental contamination during preparation. We used a case–case study based on the Swedish national *Salmonella* trawling questionnaire to identify a possible source in this outbreak. Besides timeliness, McCarthy and Gieseke proposed that the advantages of case–case studies compared with case–control studies are reduced selection and recall bias [26]. This is because outbreak cases and control cases are identified from the same surveillance system and because control cases are likely to recall their exposures better than healthy controls. Case–case studies have been used previously, both to compare sporadic cases with different gastro-intestinal diseases, to establish a baseline comparison of cases with different diseases [27,28], and in outbreak investigations [29]. In this outbreak investigation, the rationale to perform a case–case study was that crayfish is a seasonal dish, traditionally eaten in Sweden at crayfish feasts during August and September. Crayfish are typically served cold, and frozen pre-cooked crayfish are ready to eat after thawing, without reheating. Therefore, we considered a case–control study challenging because of potential recall bias among controls. Also, several confirmed cases had already responded to the questionnaire and responses from other salmonellosis cases from the same season were available. We selected control cases by date of disease onset and age to match the outbreak cases as eating habits can be expected to vary by season and between age groups. Cases linked to a large simultaneously ongoing outbreak of monophasic *Salmonella* Typhimurium was not eligible as control cases but we allowed control cases from other outbreaks only partly overlapping the season of interest. Excluding control cases linked to other outbreaks increased the aOR for crayfish but did not otherwise change the results. In this outbreak investigation, the case–case study approach was successful, identifying a suspected vehicle of infection that could be acted upon in a timely manner, and confirmed by microbiological investigation. Given the Swedish circumstances, with national trawling questionnaires in use, case–case studies could be a useful tool in future outbreaks of food and waterborne diseases. The outbreak signal detection shows the strength of a national microbial surveillance programme where *Salmonella* is timely typed for the purpose of identifying outbreaks, specifically with cases geographically widely distributed. However, the microbiological surveillance programme at PHAS only includes WGS analysis of isolates from domestic cases. Hence, it is possible that we did not detect all the outbreak cases, if they were considered being infected abroad.

## Conclusion

This investigation demonstrated the successful use of a case–case study and targeted microbiological testing to identify imported frozen, pre-cooked crayfish in dill brine as the source of the outbreak. This shows the importance of implementing and maintaining effective food safety management systems in the whole production chain, especially of ready-to-eat foods. It also shows the difficulty of completely eliminating the risk related to contaminated foods with long best-before dates, as demonstrated by the two additional cases long after the recall was made. The immediate action taken by the retail company selling the product, which included both extensive recall and analysis of food items, was important to confirm the vehicle of infection and stop the outbreak.
